# Acquired Hemophilia A: A Rare but Potentially Fatal Bleeding Disorder

**DOI:** 10.7759/cureus.5442

**Published:** 2019-08-20

**Authors:** Adeel S Yousphi, Ayesha Bakhtiar, Muhammad Arslan Cheema, Syed Nasim, Waqas Ullah

**Affiliations:** 1 Internal Medicine, Jackson Park Hospital and Medical Center, Chicago, USA; 2 Internal Medicine, Abington Memorial Hospital, Abington, USA; 3 Internal Medicine, Ross University School of Medicine, Bridgetown, BRB; 4 Internal Medicine, Abington Hospital-Jefferson Health, Abington, USA

**Keywords:** acquired hemophilia a, hematuria, factor viii, factor viii inhibitor, dysuria

## Abstract

Acquired hemophilia A is a disorder of rare entity, resulting in spontaneous bleeding in individuals with no history of bleeding disorders. It is believed to be caused by spontaneous inhibition of clotting factor VIII by autoantibodies, and is usually associated with other autoimmune conditions. The hallmark of this condition is mucocutaneous bleeding leading to ecchymosis, melena, hematoma or hematuria. Our discussion revolves around the case of an elderly male with no history of anticoagulant use presenting with hematuria. Imaging showed left kidney hemorrhage, his labs were significant for a prolonged partial thromboplastin time (PTT), and subsequent tests revealed low factor VIII levels and high factor VIII inhibitor levels, which led to the diagnosis of acquired hemophilia A in the patient. He was managed with medications resulting in normalization of factor VIII levels.

## Introduction

Acquired hemophilia A (AHA), also termed as acquired factor VIII inhibitor disorder, is a rare bleeding condition where patients with no history of bleeding disorders present with spontaneous bleeding [1]. It is a rare disease with a reported incidence of 1 per million/year, however epidemiological data may be underestimated because it remains largely undiagnosed, especially in the elderly in whom other bleeding conditions may coexist [1-2]. It can be caused by autoantibodies associated with an underlying autoimmune condition, or malignancy, however 50% of cases are idiopathic [2]. Autoimmune causes are due to the formation of factor VIII inhibiting antibodies that inhibit the clotting function of factor VIII leading to the mucosal bleeding. The bleeding associated with AHA tends to be in mucocutaneous sites or soft tissues and can lead to recurrent gastrointestinal, intramuscular, or intracranial bleeding in elderly patients as compared to hemarthrosis in younger patients with congenital hemophilia [2,3]. Diagnosis is made with laboratory tests including coagulation and mixing studies. Management classically involves control of acute bleeding episodes, treatment of underlying illness, and use of immunosuppressive medications, with an excellent outcome when diagnosed timely. This discussion highlights the importance of having a high index of suspicion for the disease, particularly in elderly patients who present with significant bleeding in the absence of risk factors such as trauma, recent surgery, anticoagulant use or known bleeding disorder.

## Case presentation

**Figure 1:**
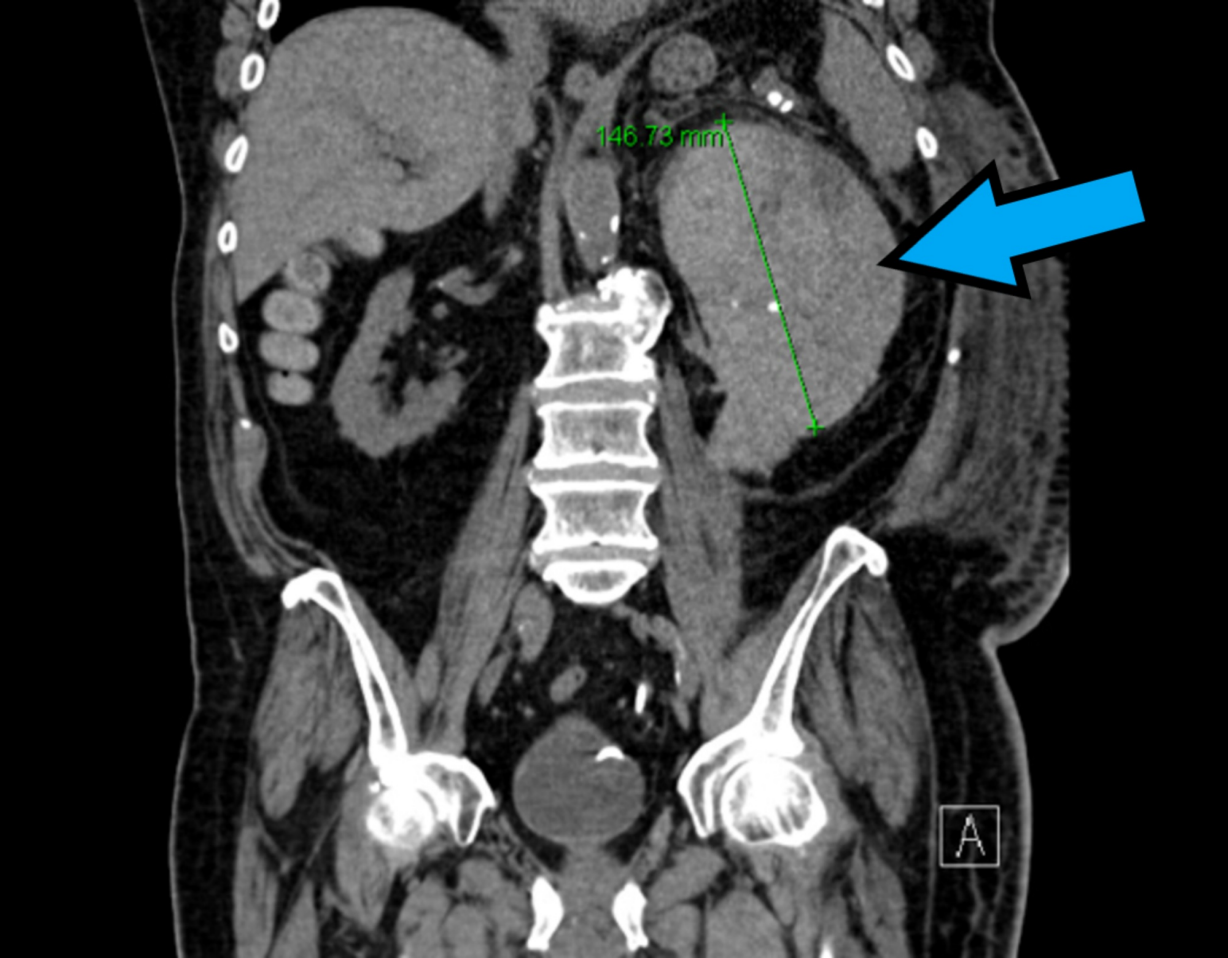
Computed tomography (CT) abdomen/pelvis showing left kidney hematoma (blue arrow)

**Figure 2:**
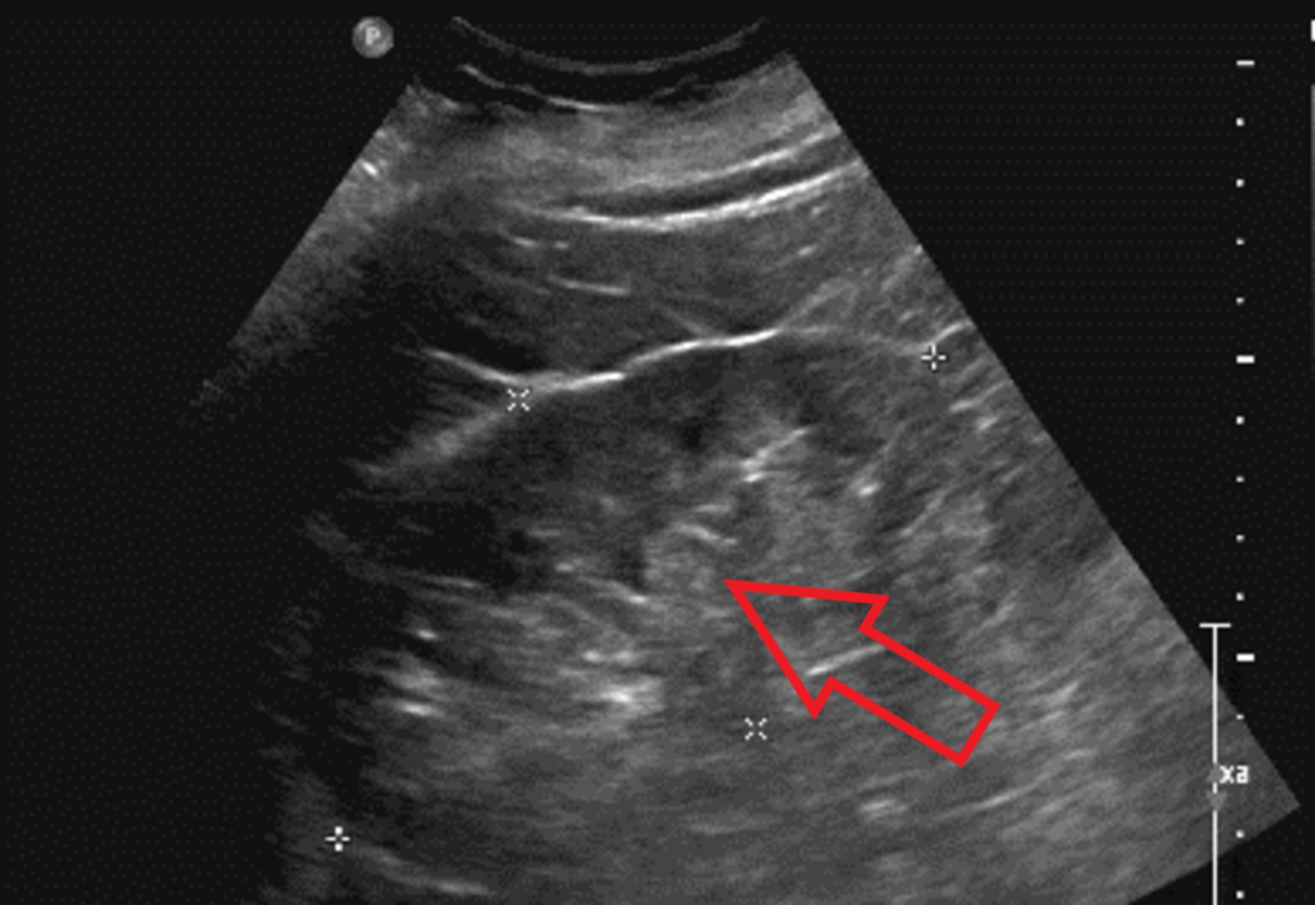
Ultrasound (US) left kidney showing echodensities (red arrow)

**Figure 3:**
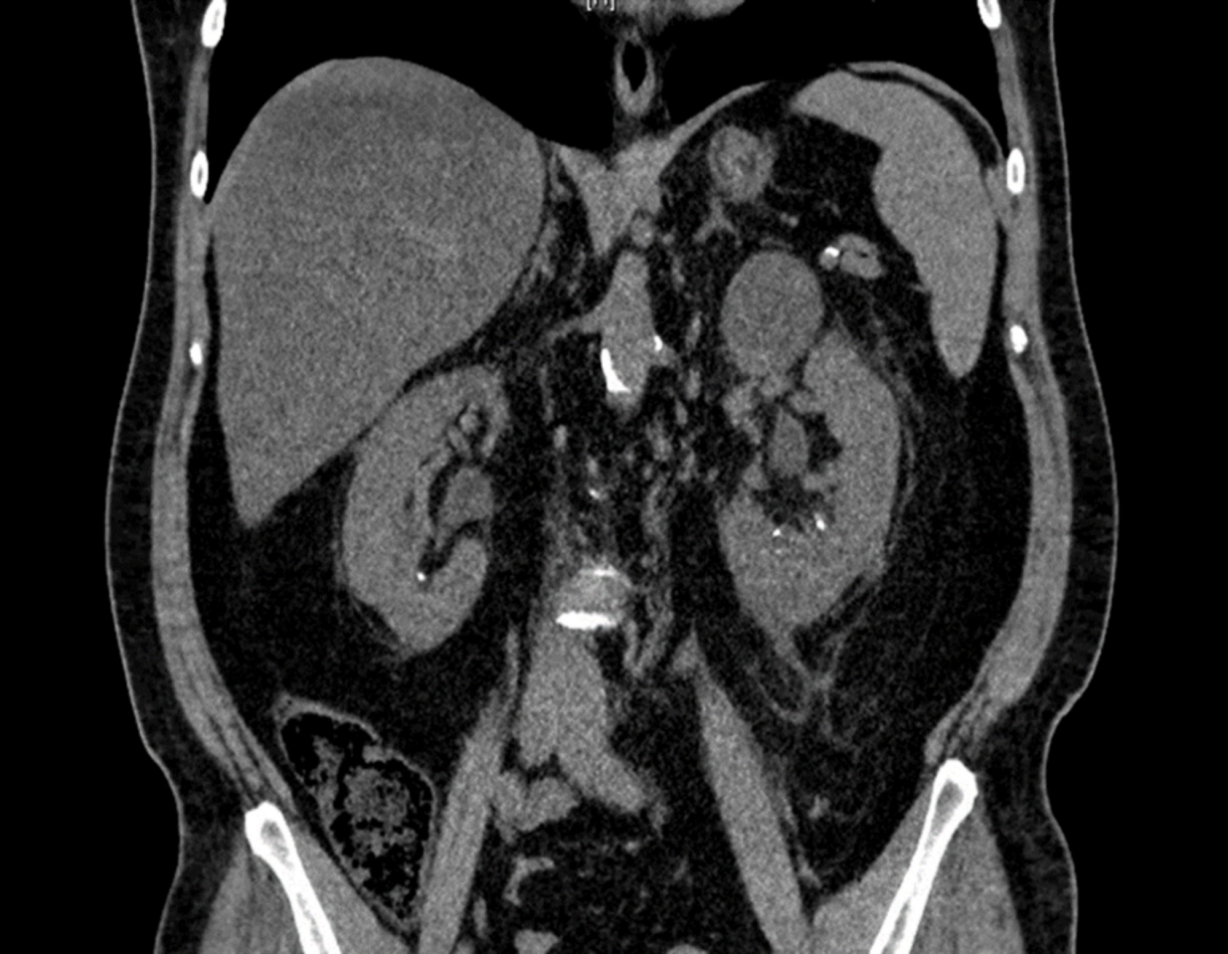
Computed tomography (CT) abdomen/pelvis showing normal kidneys at six months follow-up

An 82-year-old, hyperlipidemic, hypertensive, male came to the emergency room with complaints of dysuria and reddish discoloration of his urine for the past one month. He was not on any kind of anticoagulation therapy. Renal ultrasound and CT urogram did not show renal stones or mass. Labs showed that hemoglobin was 12.6 g/dl, international normalized ratio (INR) was 1 and partial thromboplastin time (PTT) was 42 s. Cystoscopy revealed a normal right ureteral orifice but the left ureteral orifice showed bloody efflux. There was also a significant clot burden in the renal pelvis so a stent was placed in the renal diverticulum. He complained of severe left-sided flank pain the following day, where repeat CT imaging revealed moderate hemorrhaging in the upper pole of the left kidney with difficulty in the visualization of the normal kidney (Figure 1). His renal ultrasound showed a left kidney hematoma complex with a normal right kidney (Figure 2). Repeat labs showed Hb was low (6.8 g/dl), mean corpuscular volume was 94.9 fL, platelet count was 280 x 10^9^/L and the white blood cell count was 19.4 x 10^9^/L. INR was 1 but PTT was was high (49 s). His creatinine was 1.68 mg/dL and urea nitrogen was 85 mg/dL. Serum potassium levels were high (6.5 mmol/L), whereas serum sodium levels were low (129 mmol/L). Elevated PTT prompted to check for other coagulation factors. Serum factor IX and vWF levels were within normal limits, but factor VIII levels were low. Mixing study failed to show normalization of PTT. Factor VIII inhibitor levels were significantly elevated leading to the diagnosis of acquired hemophilia A. He was started on steroids and rituximab, followed by cyclophosphamide, and the PTT normalized within four days. His discharge medications included oral steroids, and his factor VIII levels returned to normal after two weeks. Imaging performed at six months follow-up showed normal kidneys (Figure 3).

## Discussion

Acquired hemophilia is an autoimmune condition in which there is sudden production of autoantibody inhibitors in an individual without any personal or family history of bleeding [1]. The body produces autoantibodies against factor VIII in hemophila A. Since there is no previous bleeding history, the diagnosis can be missed until workup is performed. Consequently, individuals with acquired hemophilia present with massive hemorrhagic complications due to the inability of blood to clot. Often, individuals present with ecchymoses as the most common presentation. Other manifestations include muscle hematoma, melena, and hematuria and gastrointestinal bleeding [1,2]. Similarly, our case had an 82-year-old male who presented with hematuria and dysuria. In addition to the most common presentations, individuals can also present with intracranial bleeding which can be fatal; however, it is rarely seen. Unlike individuals of congenital hemophilia, bleeding into the joints is rare [2]. The bleeding can occur spontaneously or can be precipitated by events such as surgery [3,4]. The bleeding presentation of acquired hemophilia can be more severe than the bleeding seen with congenital hemophilia cases because there is a delay in diagnosing and treating the condition [1,2].

Acquired hemophilia is a disease found predominantly in the elderly population without any prior history of bleeding, such as in our case of the 82-year-old male. Additionally, no difference was seen in the distribution of the disease with regards to sex [1-3]. Researchers think that 50% of the cases are idiopathic. For the remaining 50% of cases, researchers believe an underlying disorder or infection may cause the immune system to produce antibodies against clotting factors [2]. These disorders include lupus, rheumatoid arthritis, multiple sclerosis, Sjogren's syndrome and temporal arteritis, inflammatory bowel disease, infection, diabetes, respiratory and dermatological diseases, hematological cancer, drugs such as penicillin or interferon, and pregnancy (mainly seen in the post-partum period) [2,3].

The diagnosis of acquired hemophilia A is suspected by its clinical picture confirmed by laboratory tests. Individuals present with new onset bleeding without any prior history of hemorrhagic disorders [3]. Labs will show an isolated increase in PTT with a normal PT, platelet count and thrombin time. This points to either an intrinsic pathway problem (deficient factor VIII) or a factor inhibitor [5-7]. Therefore, a mixing study must be ordered to determine if an inhibitor is present. If there is a factor deficiency, the results will show a correction of PTT [5-7]. If there is a factor inhibitor present, the PTT will remain elevated [8]. A confirmation test for factor VIII activity is performed as well to avoid misinterpretation as a lupus inhibitor [3,8]. The Bethesda assay allows us to measure the strength of the inhibitors in the plasma, expressed in Bethesda units [4].

Management of acquired hemophilia A involves treating the acute bleeding episodes, long-term management of the condition that triggered factor VIII autoantibody formation if it is present and curable, and controlling the inhibitor activity [9]. For acute hemorrhagic events, the choice of initial therapy depends on the severity of bleeding and the inhibitor titers. Desmopressin is administered to patients with low inhibitor titers and less severe bleeding [10]. Cases with severe bleeding receive human factor VIII products (recombinant factor VIII or factor VIII concentrate) if the inhibitor titers are low (<5 Bethesda units). However, activated prothrombin complex concentrate (or FEIBA, factor eight inhibitor bypassing activity), recombinant human factor VIIa, or recombinant porcine (pig) factor VIII are the treatment options if the inhibitor titers are high (i.e., ≥5 Bethesda units) [11,12]. Porcine factor VIII is more effective in cases of severe bleeding, and has much higher activity compared to human factor VIII products [13]. Eliminating the inhibitor from the body includes the use of immunosuppressive agents such as steroids, rituximab, cyclophosphamide, intravenous immunoglobulin; our patient received a combination of rituximab, steroids, and cyclophosphamide [14]. Patients can also receive prophylaxis with Emicizumab, a recombinant monoclonal antibody [15]. However, it can recur in about 20% of the cases.

Even though the disorder is rare, acquired hemophilia is a serious condition in which severe bleeding can occur in a significant number (70%) of cases and it is fatal in about five to ten percent of the cases [8,16]. The overall death rate increases to over forty percent due to multiple factors. These include delayed diagnosis, treatment inadequacies, and hemorrhagic complications during invasive procedures [8,16].

## Conclusions

Acquired hemophilia A is a rare disorder of autoimmune nature which presents as sudden bleeding, more commonly in elderly patients, without any prior trauma, anticoagulant use or known history of bleeding. Autoantibodies form against factor VIII and are seen idiopathically in 50% of the cases. Bleeding is mostly mucocutaneous or in soft tissues such as hematuria, gastrointestinal bleeding, ecchymosis or hematomas, as compared to hemarthrosis in younger patients with congenital hemophilia. The bleeding occurs spontaneously or can be triggered by events such as surgery. Coagulation and mixing studies can help in the diagnosis revealing a prolonged PTT, low factor VIII levels and high factor VIII inhibitor levels. Management includes controlling the bleeding episode and use of immunosuppressive medications, resulting mostly in a good prognosis with appropriate therapy.
